# Knockdown of LINC00665 inhibits proliferation and invasion of breast cancer via competitive binding of miR-3619-5p and inhibition of catenin beta 1

**DOI:** 10.1186/s11658-020-00235-8

**Published:** 2020-09-24

**Authors:** Minhao Lv, Qixin Mao, Juntao Li, Jianghua Qiao, Xiuchun Chen, Suxia Luo

**Affiliations:** 1 Department of Breast Surgery, The Affiliated Cancer Hospital of Zhengzhou University, Zhengzhou, Henan P.R. China; 2 Department of Medical Oncology, The Affiliated Cancer Hospital of Zhengzhou University, No. 127, Dongming Road, Jinshui District, Zhengzhou, 450008 Henan P.R. China

**Keywords:** β-Catenin, Breast cancer, *CTNNB1*, LINC00665, miR-3619-5p

## Abstract

**Background:**

Long intergenic non-protein coding RNA00665 (LINC00665) plays a crucial tumorigenic role in many cancers, such as gastric cancer and lung adenocarcinoma. However, its role and mechanism of action in the progression of breast cancer (BC) are unknown.

**Methods:**

LINC00665 expression levels were determined using quantitative polymerase chain reaction analysis with BC tissues and cell lines. BC cell proliferation was tested by performing 3-(4,5-dimethylthiazol-2-yl)-2,5-diphenyltetrazolium bromide assays, whereas BC cell migration and invasion capabilities were analyzed by performing transwell migration assays. Percentages of apoptotic cells were measured by flow cytometry. Interactions between LINC00665 and miR-3169-5p were examined by performing luciferase reporter assays, and the expression levels of proteins, such as β-catenin, were examined by western blot analysis.

**Results:**

LINC00665 was expressed at high levels in BC tissues and cells. Upregulated LINC00665 expression correlated with tumor size and tumor, node, and metastasis stages, but not with the age of patients. LINC00665 knockdown inhibited BC cell proliferation, migration, and invasion, whereas it promoted apoptosis. Moreover, bioinformatics analysis and the luciferase reporter assay revealed that LINC00665 bound the microRNA (miR) miR-3619-5p. miR-3619-5p expression correlated negatively with LINC00665 expression in BC tissues. miR-3619-5p overexpression inhibited BC cell proliferation, migration, and invasion, but promoted apoptosis. Simultaneous knockdown of LINC00665 and miR-3619-5p led to increased cell proliferation, migration, and invasion, and inhibited apoptosis. Additionally, catenin beta 1, which encodes the β-catenin protein, was the target gene of miR-3619-5p. β-catenin expression clearly decreased after LINC00665 knockdown and miR-3619-5p overexpression, but increased after simultaneous knockdown of LINC00665 and miR-3619-5p.

**Conclusion:**

LINC00665 knockdown inhibited BC cell proliferation and invasion by binding miR-3619-5p and inhibiting β-catenin expression.

## Background

Breast cancer (BC), the second most malignant tumor affecting women worldwide, has a high mortality rate [1]. Surgical resection and chemotherapy are the main modalities for treating BC, and high rates of metastasis and recurrence are responsible for the poor prognosis of these cancers. Hence, an urgent need exists for developing novel and effective therapeutic methods to improve the survival of patients with BC. BC is associated with mutations in the BRCA1, BRCA2, and p53 genes [2]. Elucidating the role of these genes would significantly help understand the molecular mechanisms underlying BC development, and may reveal specific and reliable markers for developing specific therapeutic interventions.

Long non-coding RNAs (lncRNAs) are longer than 200 nucleotides and represent a class of transcripts that are not translated into proteins [3]. lncRNAs play regulatory roles in cell proliferation, apoptosis, invasion, and metastasis in different cancers [4, 5]. One of the regulatory mechanisms involves lncRNAs acting as microRNA (miRNA) sponges by binding miRNAs and thereby modulating the post-transcriptional levels of target miRNAs [6, 7]. Many lncRNAs play important roles in BC. For example, the lncRNA myocardial infarction-associated transcript shows significantly higher expression in BC cells than in normal cells, and its knockdown inhibits BC cell proliferation [8]. Similarly, expression of the lncRNA colorectal neoplasia differentially expressed is significantly upregulated in BC cells, where it acts as a competing endogenous RNA (ceRNA) and binds miR-136, thereby activating the Wnt/catenin beta 1 (CTNNB1) signaling pathway [9]. In contrast, expression of the lncRNA maternally expressed gene 3 is remarkably downregulated in patients with BC and is negatively correlated with cancer stage and histological classification [10]. Therefore, lncRNAs modulate various physiological and pathological processes by regulating a complex network of interactions among various molecules. However, the expression pattern, functional role, underlying mechanism, and clinical significance of most annotated lncRNAs remain unclear. Therefore, exploring the functional roles of novel lncRNAs in BC may help identify new molecular targets for BC treatment.

Long intergenic non-protein coding RNA 665 (LINC00665, GenBank accession number NR_038278) plays various roles in many cancers. In hepatocellular carcinoma, LINC00665 regulates cell cycle pathways [11], whereas in lung adenocarcinoma, it acts as a ceRNA and binds miR-98, subsequently activating the aldo–keto reductase family 1 member B10 (AKR1B10)–extracellular-signal-regulated kinase (ERK)-signaling pathway [12]. In contrast, LINC00665 knockdown inhibits cell proliferation and promotes apoptosis. In gastric cancer, LINC00665 expression is related to cancer staging and poor prognosis, and accelerates cell proliferation and invasion by binding miR-149-3p [13]. However, there is a paucity of scientific literature regarding the role of LINC00665 in BC. In this study, LINC00665 expression was examined in BC tissues, and correlations between LINC00665 expression and various patient clinical characteristics were explored. Further, the effect of LINC00665 expression on BC growth was elucidated. Our results indicate that LINC00665 potentially acts as a ceRNA that binds miRNAs in BC cells.

## Methods

### Collecting clinical BC samples

This study was approved by the Ethics Committee of the Affiliated Cancer Hospital at Zhengzhou University (approval number 2019188). Informed consent forms were obtained from all participants. From June 2016 to June 2018, 106 BC tissue samples and adjacent normal tissues were obtained from patients in our hospital. None of the patients had received chemotherapy or radiotherapy before surgery. All samples were immediately transferred to and stored at − 80 °C until further analysis.

### RNA extraction, complementary DNA (cDNA) synthesis, and quantitative reverse transcription-polymerase chain reaction (qRT-PCR) analysis

The TRIzol reagent (Invitrogen) was used to extract total RNA from tumor samples. The ImProm-II Reverse Transcription System (Promega, Madison, WI, USA) was used for cDNA synthesis. Gene expression levels were analyzed by qRT-PCR on an ABI 7500 RT-PCR system (Applied Biosystems), using the SYBR Premix ExTaq Kit (Takara Bio). mRNA expression levels were normalized to 18 s rRNA expression, and miR-3619-5p expression was normalized to that of U6 RNA. Relative RNA expression levels were determined using the 2^-ΔΔCt^ method. All reactions were performed in triplicate. The sequences of the primers used in this study are as follows: LINC00665 (NR_038278), forward: 5′-GGTGCAAAGTGGGAAGTGTG-3′ and reverse: 5′-CGGTGGACGGATGAGAAACG-3′; 18 s rRNA, forward: 5′-CCTGGATACCGCAGCTAGGA-3′ and reverse: 5′-GCGGCGCAATACGAATGCCCC-3′; miR-3619-5p, forward: 5′-UCAGCAGGCAGGCUGGUGCAGC-3′ and reverse: 5′-GCUGCACCAGCCUGCCUGCUGA-3′; U6, forward: 5′-CTCGCTTCGGCAGCACATATACTA-3′ and reverse: 5′-ACGAATTTGCGTGTCATCCTTGCG-3′.

### Cell culture

BC cell lines (MDA-MB-231 and MCF-7) and normal human breast epithelial cells (MCF-10A) were purchased from the American Type Culture Collection (Manassas, VA, USA). The cells were cultured according to the methods provided by the supplier.

### Plasmids, cell transfection, and luciferase activity assay

Plasmids used to knock down LINC00665 (si-LINC00665–1, si-LINC00665–2, and si-LINC00665–3) and the negative control plasmid (si-NC) were purchased from Gene Pharma (Shanghai). miRNA mimics and inhibitors (miR-3619-5p mimic, miR-3619-5p inhibitor, miR-NC mimic, and miR-NC inhibitor) were purchased from RiboBio (Guangzhou). All plasmids were transfected using Lipofectamine 2000 (Invitrogen). The miRNA-binding sites of LINC00665 were predicted using the StarBase 3.0 and miRcode software programs. The wild-type (WT) 3′-untranslated region (UTR) of LINC00665 or *CTNNB1* with putative binding sites for miR-3619-5p, or mutant (MUT) 3′-UTRs for each site, were cloned into the psi-CHECK-2 plasmid (Promega) to construct the luciferase reporters WT-LINC00665 and MUT-LINC00665. Luciferase reporter plasmids and the miR-3619-5p or NC mimics were co-transfected into BC cells to analyze the effect of miR-3619-5p on the luciferase activity. After transfection for 48 h, the activities of firefly luciferase (F) and Renilla luciferase (R) were measured using the Dual-Luciferase Reporter Assay System (Promega). The relative luciferase activity of each group was calculated based on the R/F ratio.

### Cell proliferation and apoptosis assays

Cell proliferation and apoptosis analyses were performed at 48 h after transfection. Cell proliferation was analyzed using the 3-(4,5-dimethylthiazol-2-yl)-2,5-diphenyltetrazolium bromide (MTT) assay (Sigma, USA), whereas apoptotic cells were measured using an Annexin V-FITC Apoptosis Detection Kit (Keygentec, China) and a flow cytometer (BD Biosciences), following the manufacturers’ recommended protocols. All experiments were performed in triplicate.

### Transwell migration and cell invasion assays

Twenty-four well transwell plates (BD Biosciences, CA) were used for the cell migration and cell invasion assays. For the invasion assays, a thin layer of Matrigel (BD Biosciences) was used to cover the inner surface of the membrane, whereas the membrane without Matrigel was used for the cell migration assays. Briefly, transfected MCF-7 or MD-MBA-231 cells (2 × 10^5^) in 200 μL of serum-free medium were seeded into the top chamber, and 600 μL of culture medium with 20% serum was added into the lower chamber. Twenty-four hours later, the migrated or invaded cells were fixed using methanol and stained with 0.5% crystal violet solution. Stained cells were visualized and counted using microscopy (Olympus, Tokyo, Japan). All experiments were conducted in triplicate.

### Western blot assay

Total protein was extracted from harvested cells using radioimmunoprecipitation assay buffer. After separation by sodium dodecyl sulfate-polyacrylamide gel electrophoresis, the proteins were transferred to a nitrocellulose membrane. After blocking, the membranes were incubated at 4 °C overnight with primary antibodies against cyclin D1 (ab16663, 1:200), cleaved caspase-3 (ab13847, 1:500), cleaved caspase-9 (ab2324, 1:500), matrix metalloproteinase (MMP)-2 (ab92536, 1:1000), MMP-9 (ab38898, 1:1000), β-catenin (ab16051, 1:4000), or glyceraldehyde 3-phosphate dehydrogenase (ab181602, 1:10000) (Abcam, Cambridge, UK, USA), followed by incubation with horseradish peroxidase-labeled goat anti-rabbit IgG antibody (ab205718, 1:20,000) for 2 h at 25 °C. After washing three times with Tris-buffered saline and 0.1% Tween 20, the protein bands were detected using an Enhanced Chemiluminescence Detection Kit (Beyotime) and the signals were exposed to X-ray film. All experiments were performed independently, in triplicate.

### Statistical analysis

Experimental data were analyzed using SPSS 19.0 statistical software (IBM, Inc.). The data shown are presented as the mean ± standard deviation. Student’s *t*-test or one-way analysis of variance was performed to analyze statistical differences between groups. *P* < 0.05 was considered to reflect a significant difference.

## Results

### LINC00665 was overexpressed in BC tissues

**Fig. 1:**
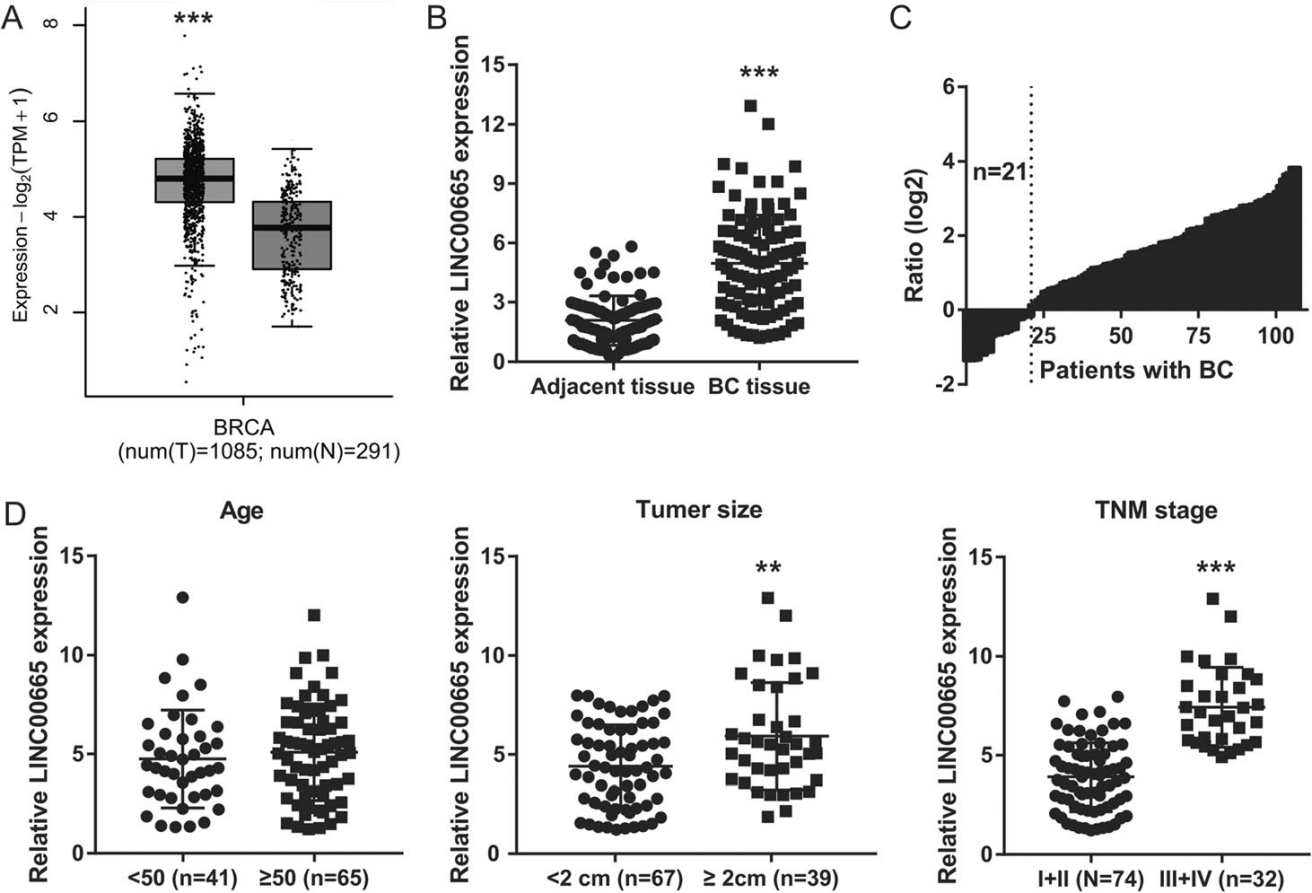
LINC00665 upregulation in breast cancer (BC) tissues and associations with different patient clinical characteristics. **a** LINC00665 expression levels in BC tissues (*n* = 1085) and normal breast tissues, as analyzed using gene expression profiling interactive analysis (GEPIA, *n* = 291). **b** LINC00665 expression in BC tissues and adjacent normal breast tissues, as measured using quantitative reverse transcription-polymerase chain reaction (qRT-PCR). **c** The number of BC patients (x-axis) versus the distribution plot of the log2 values of the relative expression ratio of LINC00665 in BC tissues to that in adjacent normal breast tissues (y-axis). **d** Relationships between LINC00665 expression and different patient clinical characteristics. **P < 0.01, ***P < 0.001

The expression profile of the LINC00665 gene was analyzed using the Gene Expression Profiling Interactive Analysis (GEPIA) method with BC and normal tissues. LINC00665 gene expression was significantly upregulated in BC tissues (Fig. 1a). Further, LINC00665 expression was remarkably high in all tumor samples, as determined by qRT-PCR analysis (Fig. 1 b, c). LINC00665 expression was significantly associated with the tumor size and the tumor, node, and metastasis (TNM) stage, but not with the age of the patients (Fig. 1d).

### LINC00665 expression was inhibited after transfection with the si-LINC00665 plasmid

**Fig. 2:**
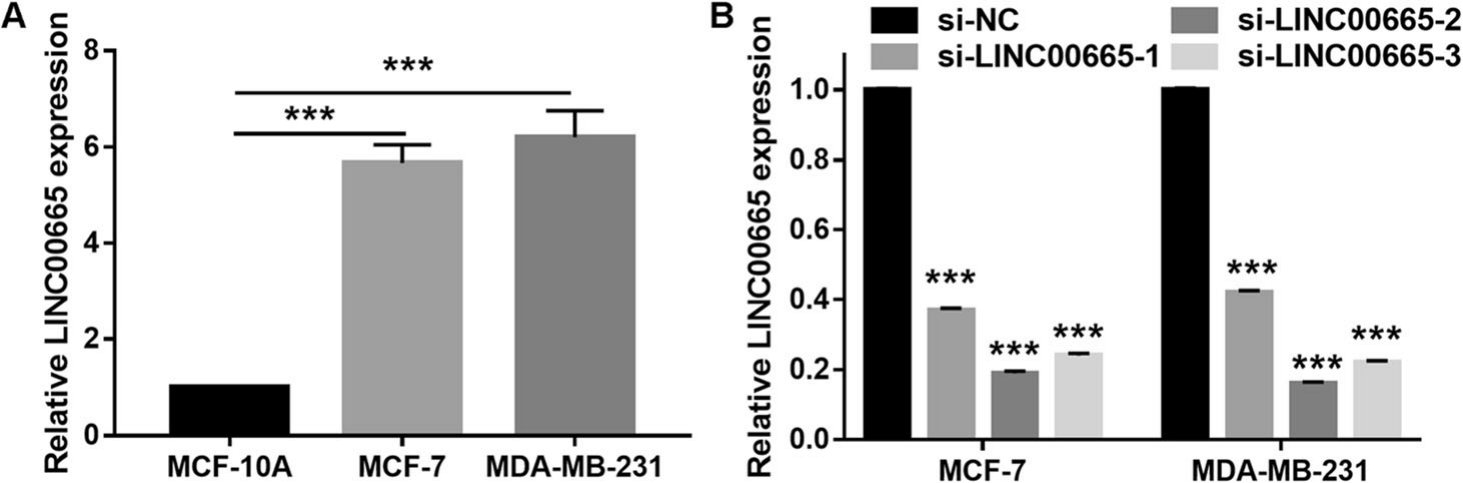
LINC00665 expression in breast cancer (BC) cells. **a** LINC00665 knockdown efficiency in BC cells and in normal cells, as measured by performing quantitative reverse transcription-polymerase chain reaction (qRT-PCR) analysis. **b** LINC00665 knockdown efficiency, as measured by qRT-PCR, 48 h after transfection. ***P < 0.001

The results of qRT-PCR analysis showed that LINC00665 expression levels were significantly higher in BC cells than in the normal breast cell line (MCF-10A) (Fig. 2a). To study the biological function of LINC00665 in BC cells in vitro, LINC00665 expression was knocked down by transfecting cells with the si-LINC00665–1, si-LINC00665–2, and si-LINC00665–3 plasmids; cells transfected with the si-NC plasmid served as the control. The knockdown efficiency was confirmed by qRT-PCR, which showed that LINC00665 expression was significantly lower in cells transfected with the si-LINC00665 plasmids than in the si-NC transfectants; moreover, si-LINC00665–02 could knock down LINC00665 expression the most efficiently (Fig. 2b).

### Effects of LINC00665 silencing on BC cells

**Fig. 3:**
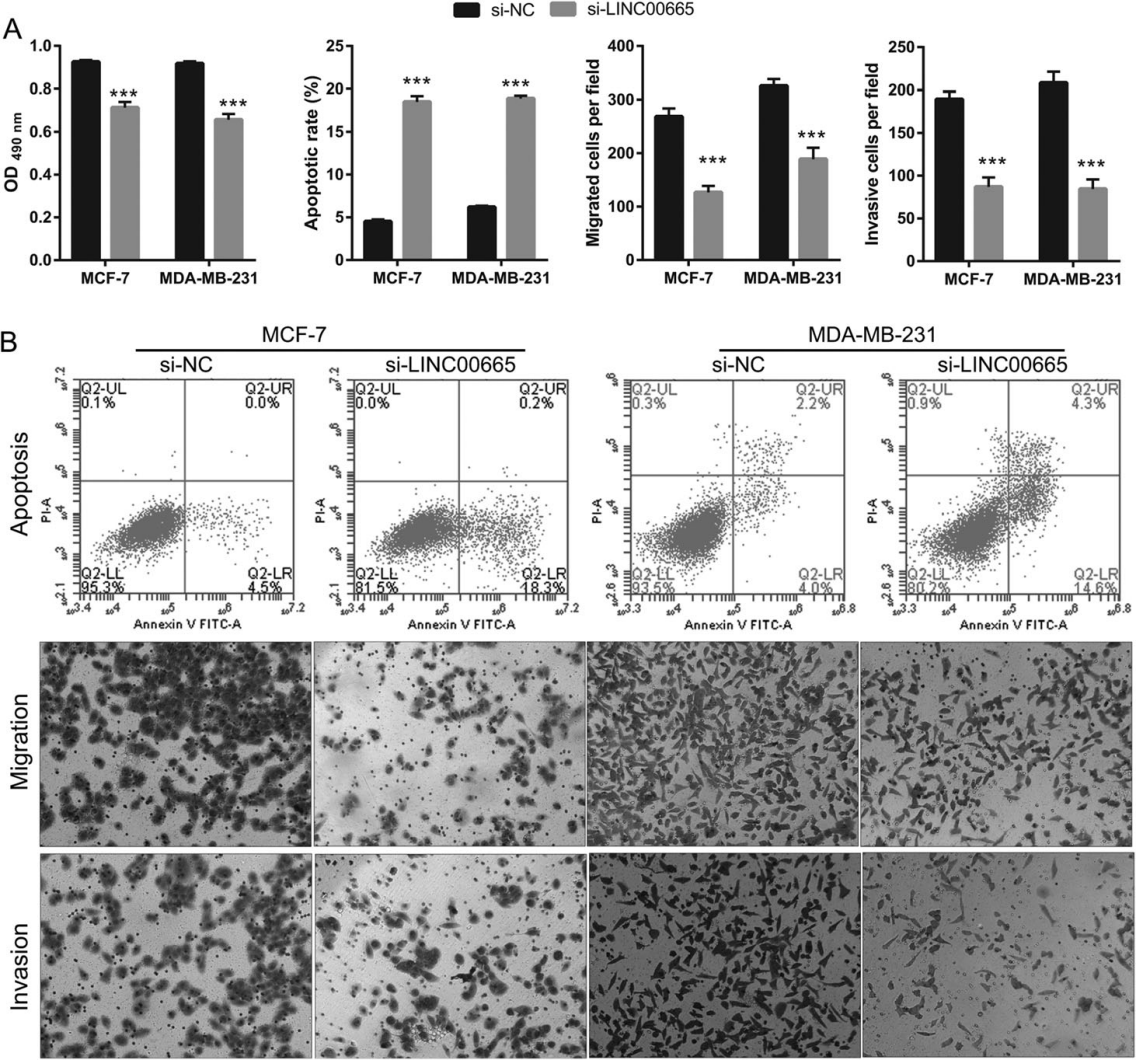
LINC00665 knockdown inhibited breast cancer (BC) cell proliferation, migration, and invasion, but promoted apoptosis. **a** BC cell proliferation, apoptosis, migration, and invasion were measured by performing 3-(4,5-dimethylthiazol-2-yl)-2,5-diphenyltetrazolium bromide (MTT) assays, flow cytometry, transwell migration assays, and invasion assays, respectively. The data shown are the mean ± standard deviation (SD). **b** Representative images showing cell apoptosis, migration, and invasion. ***P < 0.001

The proliferation, migration, and invasion properties of the BC cells transfected with si-LINC00665 plasmids for 48 h were analyzed. LINC00665 silencing markedly compromised BC cell proliferation, migration, and invasion, when compared to cells transfected with the si-NC plasmid; in contrast, LINC00665 silencing significantly increased the percentage of apoptotic cells (Fig. 3). Additionally, LINC00665 knockdown did not significantly affect the proliferation of MCF-10A cells (Fig. S1). Finally, compared to the si-NC treatment group, LINC00665 silencing clearly reduced the protein expression levels of cyclin D1, MMP-2, and MMP-9, but enhanced the expression levels of cleaved caspase-3 and cleaved caspase-9 (Fig. S2).

### miR-3619-5p was a direct binding target of LINC00665

**Fig. 4:**
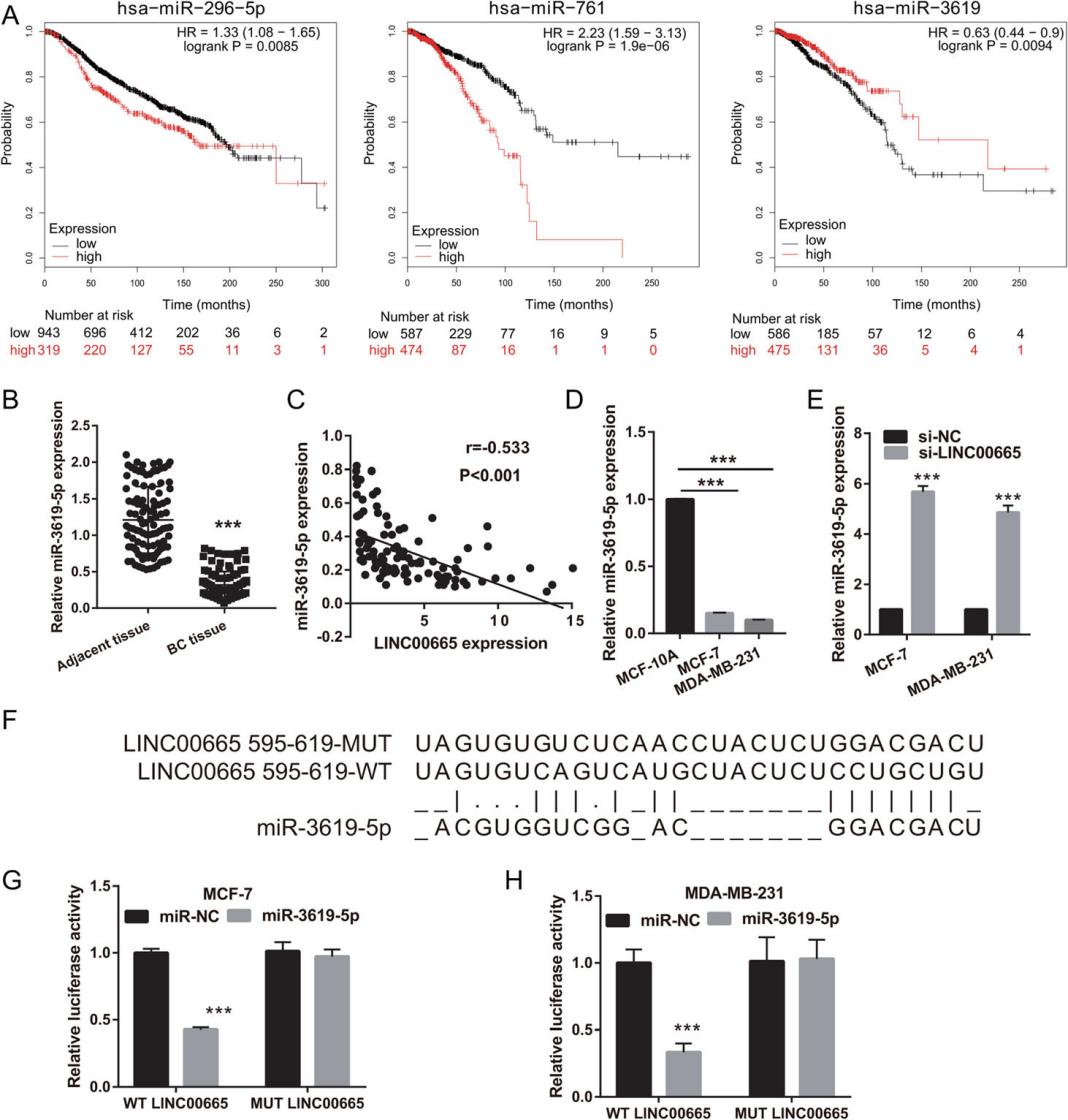
miR-3619-5p is a direct binding target of LINC00665. **a** The relationships of miR-3619-5p, miR-761, and miR-296-3p expression with poor patient prognosis were analyzed using the Kaplan–Meier plotter (http://kmplot.com/analysis/index.php). **b** miR-3619-5p expression levels in breast cancer (BC) tissues. **c** Relationship between expression levels of LINC00665 and miR-3619-5p. **d** miR-3619-5p expression levels in BC cells. **e** miR-3619-5p expression levels in BC cells after LINC00665 was knocked down by transfecting cells with the si-LINC00665 plasmid. **f** Potential binding sites between LINC00665 and miR-3619-5p, as predicted using the LncBase Predicted v.2 website. **g** and **h** Luciferase reporter assays showing that LINC00665 has a direct binding site for miR-3619-5p. ***P < 0.001

**Fig. 5:**
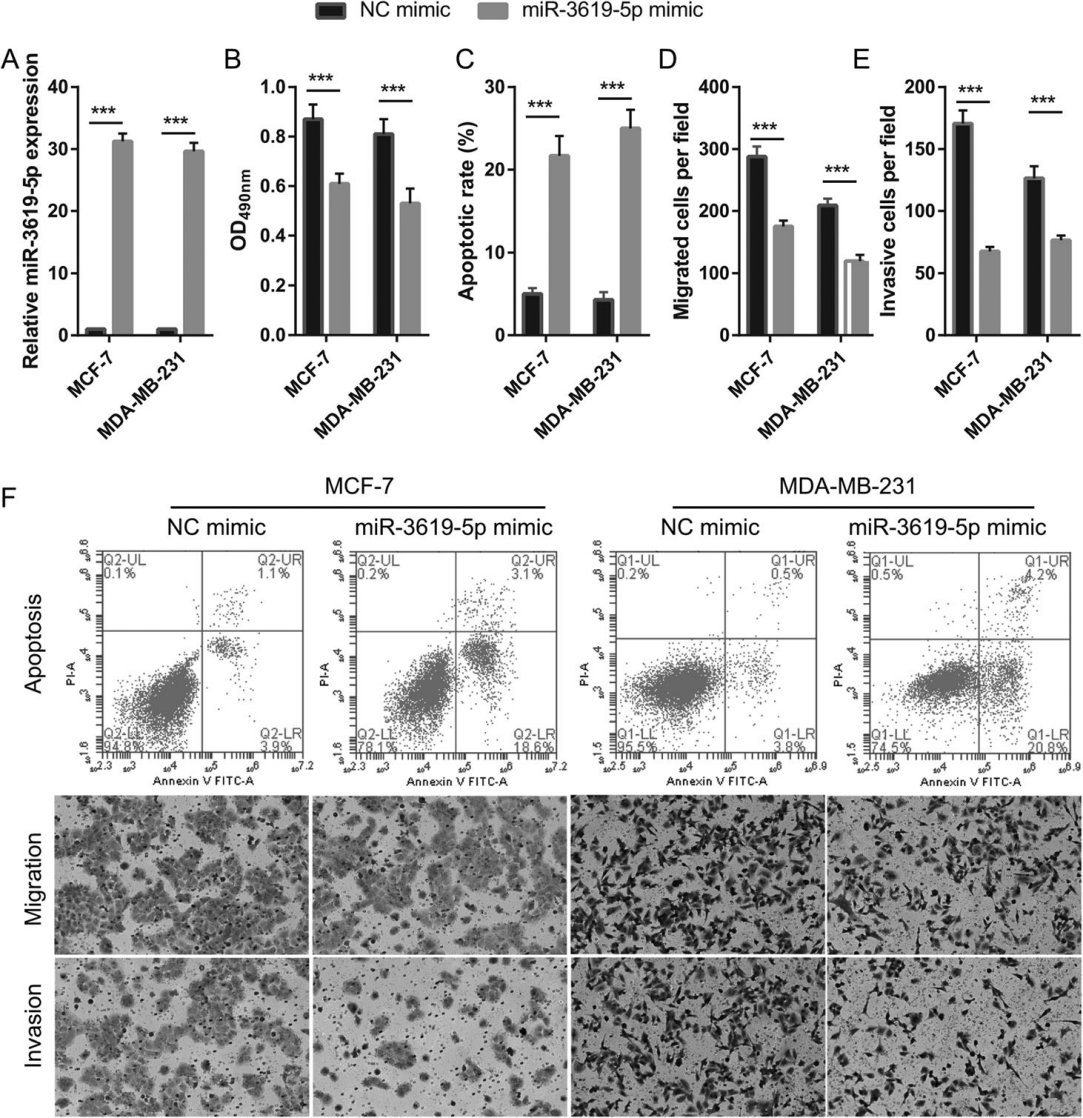
miR-3619-5p overexpression inhibited proliferation, migration, and invasion of breast cancer (BC) cells, but promoted apoptosis. **a** miR-3619-5p expression, as measured by quantitative reverse transcription-polymerase chain reaction (qRT-PCR) analysis at 48 h after transfecting the miR-3619-5p mimic. **b**–**e** Proliferation, apoptosis, migration, and invasion of BC cells measured by performing 3-(4,5-dimethylthiazol-2-yl)-2,5-diphenyltetrazolium bromide (MTT) assays, flow cytometry, transwell migration assays, and invasion assays, respectively. The data shown are the mean ± standard deviation (SD). **f** Representative images showing cell apoptosis, migration, and invasion. ***P < 0.001

**Fig. 6:**
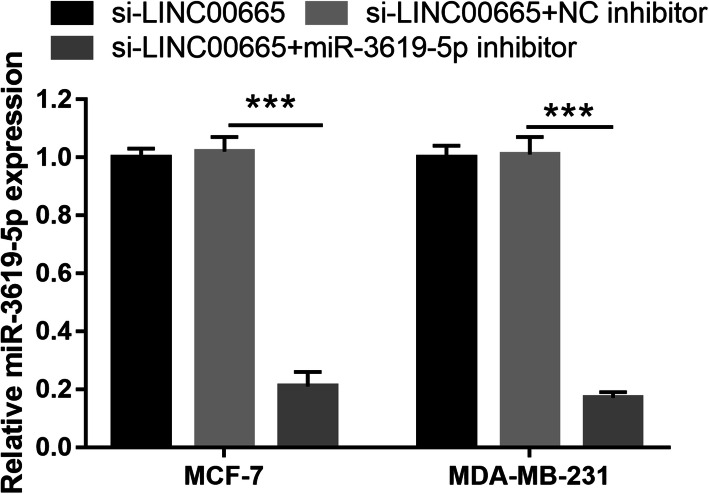
miR-3619-5p expression was significantly inhibited after simultaneous transfection of the si-LINC00665 plasmid and the miR-3619-5p inhibitor. miR-3619-5p expression, as measured by quantitative reverse transcription-polymerase chain reaction (qRT-PCR) after simultaneous transfection of the si-LINC00665 plasmid and the miR-3619-5p (or NC) inhibitor at 48 h. ***P < 0.001

**Fig. 7:**
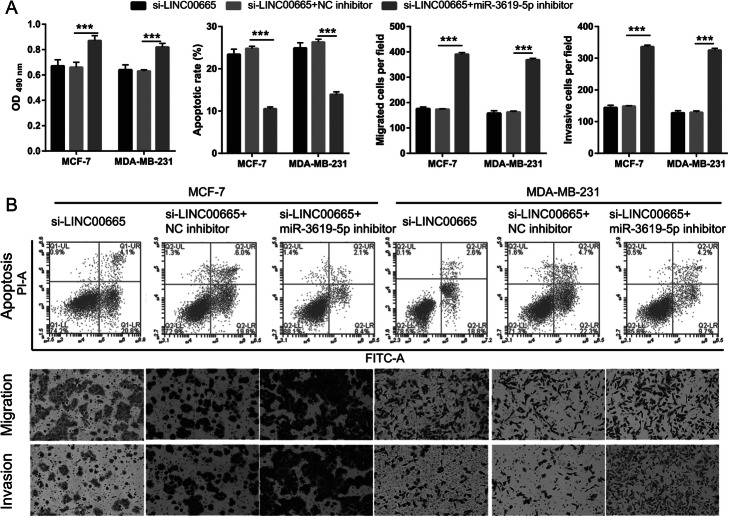
Simultaneous knockdown of LINC00665 and miR-3619-5p promoted breast cancer (BC) cell proliferation, migration, and invasion, but inhibited apoptosis. **a** Proliferation, apoptosis, migration, and invasion, as measured by performing 3-(4,5-dimethylthiazol-2-yl)-2,5-diphenyltetrazolium bromide (MTT) assays, flow cytometry, transwell migration assays, and invasion assays, respectively. The data shown are the mean ± standard deviation (SD). **b** Representative images showing BC cell apoptosis, migration, and invasion. ***P < 0.001

The LncBase Predicted v.2 website was used to predict (threshold ≥0.6) potential binding targets of LINC00665, including miR-3619-5p, miR-761, and miR-296-3p. These data were analyzed using the Kaplan–Meier plotter (http://kmplot.com/analysis/index.php) and showed that miR-3619-5p expression was significantly inhibited (Fig. 4a). We hypothesized that LINC00665 could act as a ceRNA to bind miR-3619-5p in BC cells. Hence, miR-3619-5p was further characterized in BC. miR-3619-5p expression levels were considerably lower in BC tissues than in normal tissues (Fig. 4b). Furthermore, a negative correlation was observed between LINC00665 and miR-3619-5p expression (Fig. 4c). The expression levels of miR-3619-5p were remarkably lower in MCF-7 and MDA-MB-231 cells (Fig. 4d). LINC00665 knockdown significantly increased miR-3619-5p expression in MCF-7 and MDA-MB-231 cells (Fig. 4e), but did not significantly affect miR-761 and miR-296-3p expression (Fig. S3). Potential binding sites between LINC00665 and miR-3619-5p were predicted using the LncBase Predicted v.2 website (Fig. 4f). Significantly lower luciferase activity was observed in cells co-transfected with WT-LINC00665 and the miR-3619-5p mimic, whereas no inhibitory effect was observed in cells co-transfected with MUT-LINC00665 and the miR-3619-5p mimic (Fig. 4g, h). Finally, the abilities of BC cells to proliferate, migrate, and invade decreased significantly following miR-3619-5p overexpression, whereas apoptosis significantly increased (Fig. 5). Compared to the NC mimic-treated group, the miR-3619-5p-overexpressing group had clearly reduced protein expression levels of cyclin D1, MMP-2, and MMP-9, but enhanced cleaved caspase-3 and cleaved caspase-9 expression (Fig. S4). miR-3619-5p overexpression did not significantly affect the proliferation of MCF-10A cells (Fig. S1). To elucidate the biological relationship between LINC00665 and miR-3619-5p, simultaneous knockdown of LINC00665 and miR-3619-5p was performed in MCF-7 and MDA-MB-231 cells. miR-3619-5p expression was significantly inhibited in cells simultaneously transfected with si-LINC00665 and the miR-3619-5p inhibitor, compared to cells simultaneously transfected with si-LINC00665 and the NC inhibitor (Fig. 6). The simultaneous knockdown of LINC00665 and miR-3619-5p enhanced the proliferation, migration, and invasion of MCF-7 and MDA-MB-231 cells, while reducing apoptosis in both the cell lines (Fig. 7). Additionally, compared to the group treated with si-LINC00665 and the NC inhibitor, simultaneous knockdown of LINC00665 and miR-3619-5p enhanced the protein expression levels of cyclin D1, MMP-2, and MMP-9, but reduced the expression levels of cleaved caspase-3 and cleaved caspase-9 (Fig. S4).

### LINC00665 and miR-3619-5p regulated β-catenin expression

**Fig. 8:**
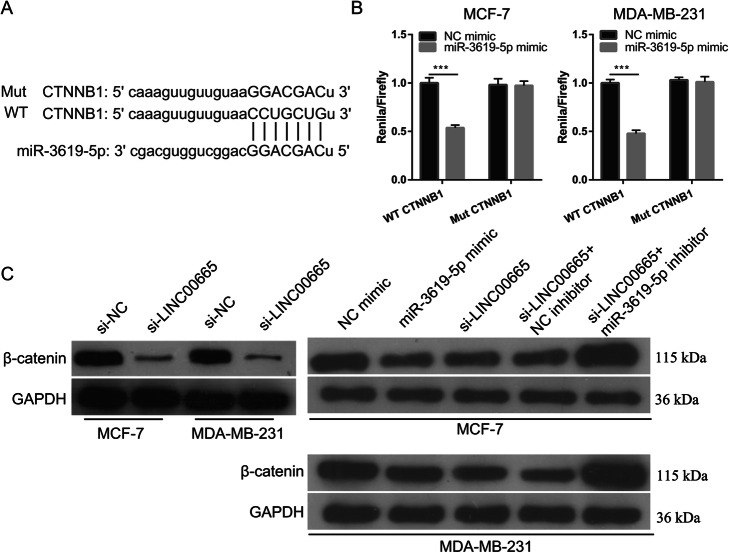
Effect of LINC00665/miR-3619-5p expression on β-catenin expression in breast cancer (BC) cells. **a** Potential binding sites between *CTNNB1* and miR-3619-5p. **b** Luciferase reporter assay showing that the *CTNNB1* 3′-untranslated region has a direct binding site for miR-3619-5p. **c** Western blot showing LINC00665 knockdown and that miR-3619-5p overexpression inhibited expression of β catenin (encoded by *CTNNB1*). Simultaneous knockdown of LINC00665 and miR-3619-5p promoted β-catenin expression in MCF-7 and MDA-MB-231 cells

Previous data showed that miR-3619-5p silences the expression of *CTNNB1* (which encodes the β-catenin protein) and inhibits the Wnt/CTNNB1 pathway [14]. Bioinformatics analysis and luciferase reporter assays revealed that the *CTNNB1* 3′-UTR was a target of miR-3619-5p (Fig. 8a). LINC00665 knockdown and miR-3619-5p overexpression in MDA-MB-231 cells clearly decreased β-catenin protein expression (Fig. 8c).

## Discussion

BC is one of the most common malignancies affecting women and has a complex and heterogeneous etiology. To date, the regulatory mechanisms and signaling pathways underlying BC initiation and progression remain unclear. In this study, the expression of lncRNAs and their roles in BC were evaluated. The results demonstrated that LINC00665 acted as a miR-3619-5p sponge and inhibited tumorigenesis by modulating β-catenin expression.

Earlier reports showed that LINC00665 plays an important role in the progression of various tumors. In this study, LINC00665 expression was highly upregulated in BC tissues and cells. Further, knocking down LINC00665 inhibited BC cell proliferation, migration, and invasion, while promoting apoptosis, and these observations were consistent with those of previous studies. For example, in hepatocellular carcinoma [15], LINC00665 expression is remarkably upregulated, and its knockdown inhibits cancer cell viability and induces apoptosis and autophagy by binding miR-186-5p. Similarly, in non-small-cell lung cancer (NSCLC) [16], LINC00665 expression is significantly upregulated and its silencing suppresses cell proliferation, induces apoptosis, and modulates the phosphoinositide 3-kinase/protein kinase B-signaling pathway. Additionally, we found that LINC00665 expression positively correlated with tumor size and TNM stage, but not with the age of patients; these results are consistent with earlier results related to gastric cancer [13], wherein LINC00665 expression correlated with the TNM stage and a poor patient prognosis. These results suggest that LINC00665 knockdown inhibits BC proliferation and invasion.

Accumulating evidence indicates that a common biological function of lncRNAs is to act as a ceRNA to competitively bind miRNAs [17] and regulate mRNA expression at the post-transcriptional level. Data from an earlier study on lung adenocarcinoma [12] showed that miR-98 expression negatively correlated with LINC00665 expression and that the latter acted as a ceRNA to competitively bind miR-98, thereby activating the AKR1B10–ERK-signaling pathway to facilitate cell proliferation and metastasis. Bioinformatics analysis indicated that LINC00665 contains a direct binding site for miR-3619-5p. In this study, miR-3619-5p expression was significantly downregulated in BC tissues and cells; similar observations were made previously. For instance, in NSCLC [18], miR-3619-5p expression was significantly downregulated, and miR-3619-5p overexpression inhibited β-catenin protein expression (without affecting the corresponding mRNA levels), as well as cell growth and invasion. Similarly, in prostate cancer [19], miR-3619-5p expression was markedly downregulated and induced cell cycle arrest and inhibited cell proliferation. These studies also suggest that miR-3619-5p acts as an anti-cancer gene. Similar to those studies, miR-3619-5p overexpression inhibited BC proliferation, migration, and invasion; thus, it acted as an anti-cancer gene.

In this study, a negative correlation between miR-3619-5p and LINC00665 expression was observed in BC cells, indicating that LINC00665 might function as a molecular sponge for miR-3619-5p. Knocking down LINC00665 and miR-3619-5p simultaneously in BC cells led to increased cell proliferation, migration, and invasion, while apoptosis was inhibited. These observations contrasted with the results obtained when LINC00665 alone was knocked down, suggesting that LINC00665 knockdown inhibited BC proliferation and invasion by competitively binding miR-3619-5p.

*CTNNB1* is the key downstream effector and transcriptional co-activator of T-cell factor/lymphoid enhancer factor target gene expression in the Wnt/β-catenin pathway. *CTNNB1* is located on chromosome 3p21 and encodes the β-catenin protein. *CTNNB1* expression is abnormally increased in BC, which suggests its use as a potential therapeutic target in BC [20, 21]. In this study, the *CTNNB1* 3′-UTR was found to be the target of miR-3619-5p. In a related study, miR-3619-5p overexpression in bladder cancer was shown to inhibit tumorigenesis by targeting the *CTNNB1* 3′-UTR and inhibiting β-catenin expression [14]. In this study, β-catenin expression was inhibited by LINC00665 knockdown and miR-3619-5p overexpression, but was clearly increased by simultaneous knockdown of LINC00665 and miR-3619-5p. Collectively, these results demonstrate that LINC00665 exerted an anti-tumorigenic effect by interacting with miR-3619-5p to target the CTNNB1 3′-UTR and regulate β-catenin expression.

## Conclusions

Knocking down LINC00665 expression inhibited the proliferation, migration, and invasion of BC cells. Knocking down LINC00665 expression also inhibited β-catenin expression by competitively binding miR-3619-5p in BC cells. These results suggest that LINC00665 and miR-3619-5p could serve as novel and reliable targets for developing effective BC treatment modalities.

## Supplementary information

**Additional file 1: Figure S1.** LINC00665 knockdown and miR-3619-5p overexpression did not significantly affect MCF-10A cell proliferation. (A and B) LINC00665 and miR-3619-5p expression levels were measured by quantitative reverse transcription-polymerase chain reaction (qRT-PCR), at 48 h after transfection. (C) Proliferation of MCF-10A cells was measured by performing 3-(4,5-dimethylthiazol-2-yl)-2,5-diphenyltetrazolium bromide (MTT) assays at 48 h after transfection.

**Additional file 2: Figure S2.** LINC00665 silencing reduced the protein expression levels of cyclin D1, matrix metalloproteinase (MMP)-2, and MMP-9 expression, while enhancing cleaved caspase-3 and cleaved caspase-9 expression, as measured by western blot analysis.

**Additional file 3: Figure S3.** LINC00665 silencing did not significantly affect miR-761 and miR-296-3p expression. miR-761 and miR-296-3p expression, as measured by quantitative reverse transcription-polymerase chain reaction (qRT-PCR) analysis at 48 h after transfection with the si-LINC00665 plasmid.

**Additional file 4: Figure S4.** Effects of miR-3619-5p and LINC00665 on expression levels of cyclin D1, matrix metalloproteinase (MMP)-2, MMP-9, cleaved caspase-3, and cleaved caspase-9. The protein expression levels were measured by performing western blot analysis.

## Data Availability

The data that support the findings of this study are available from the corresponding author upon reasonable request.

## Abbreviations

BC: Breast cancer
ceRNA: Competing endogenous RNA
GEPIA: Gene expression profiling interactive analysis
LINC00665: Long intergenic non-protein coding RNA00665
lncRNA: Long non-coding RNA
miRNA: microRNA
MMP: Matrix metalloproteinase
MTT: 3-(4,5-dimethylthiazol-2-yl)-2,5-diphenyltetrazolium bromide
NC: Negative control
qRT-PCR: quantitative reverse transcription-polymerase chain reaction
ST: Standard deviation
3′-UTR: 3′-untranslated region
