# Supplementary figures and images for: Knockdown of LINC00665 inhibits proliferation and invasion of breast cancer via competitive binding of miR-3619-5p and inhibition of catenin beta 1
Source: Cell Mol Biol Lett. 2020 Sep 24;25:43. doi: 10.1186/s11658-020-00235-8 (PMC7513511; doi:10.1186/s11658-020-00235-8)

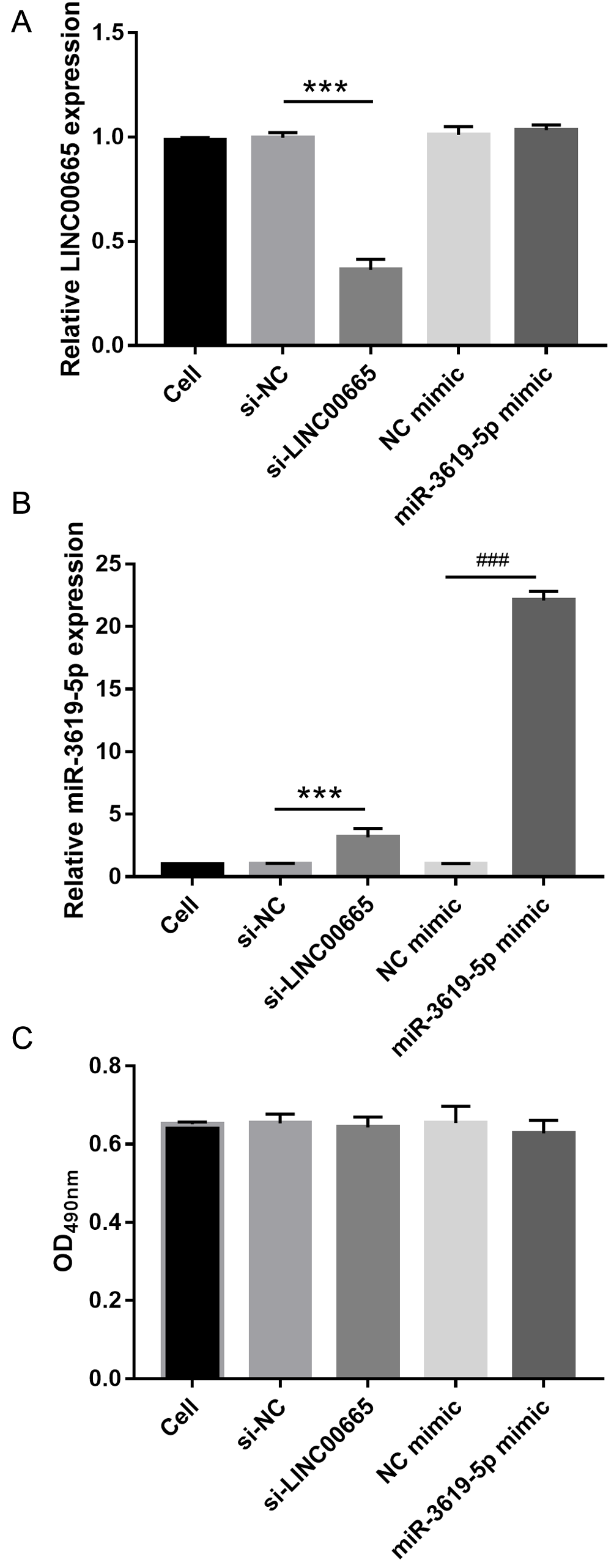

Supplement: Supplementary file 1 — Additional file 1: Figure S1. LINC00665 knockdown and miR-3619-5p overexpression did not significantly affect MCF-10A cell proliferation. (A and B) LINC00665 and miR-3619-5p expression levels were measured by quantitative reverse transcription-polymerase chain reaction (qRT-PCR), at 48 h after transfection. (C) Proliferation of MCF-10A cells was measured by performing 3-(4,5-dimethylthiazol-2-yl)-2,5-diphenyltetrazolium bromide (MTT) assays at 48 h after transfection. [file 11658_2020_235_MOESM1_ESM.tif]

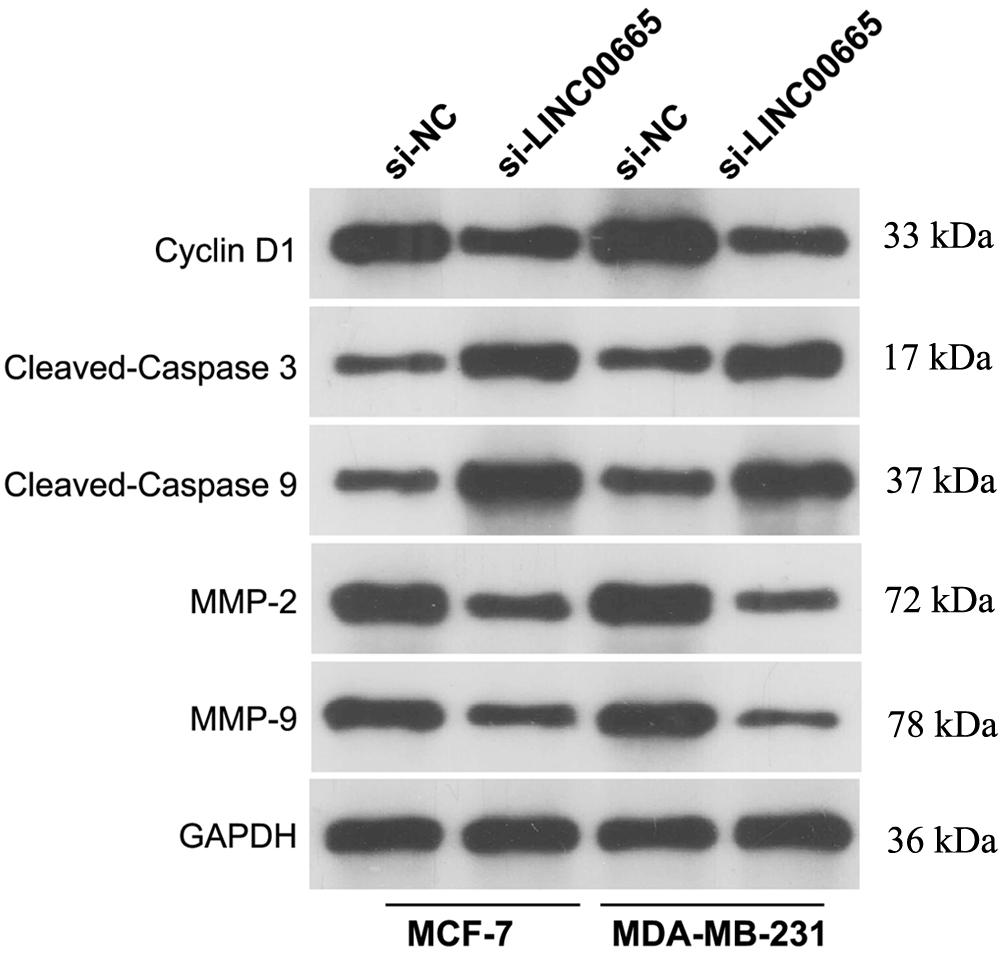

Supplement: Supplementary file 2 — Additional file 2: Figure S2. LINC00665 silencing reduced the protein expression levels of cyclin D1, matrix metalloproteinase (MMP)-2, and MMP-9 expression, while enhancing cleaved caspase-3 and cleaved caspase-9 expression, as measured by western blot analysis. [file 11658_2020_235_MOESM2_ESM.zip › R2-Figure S2.tif]

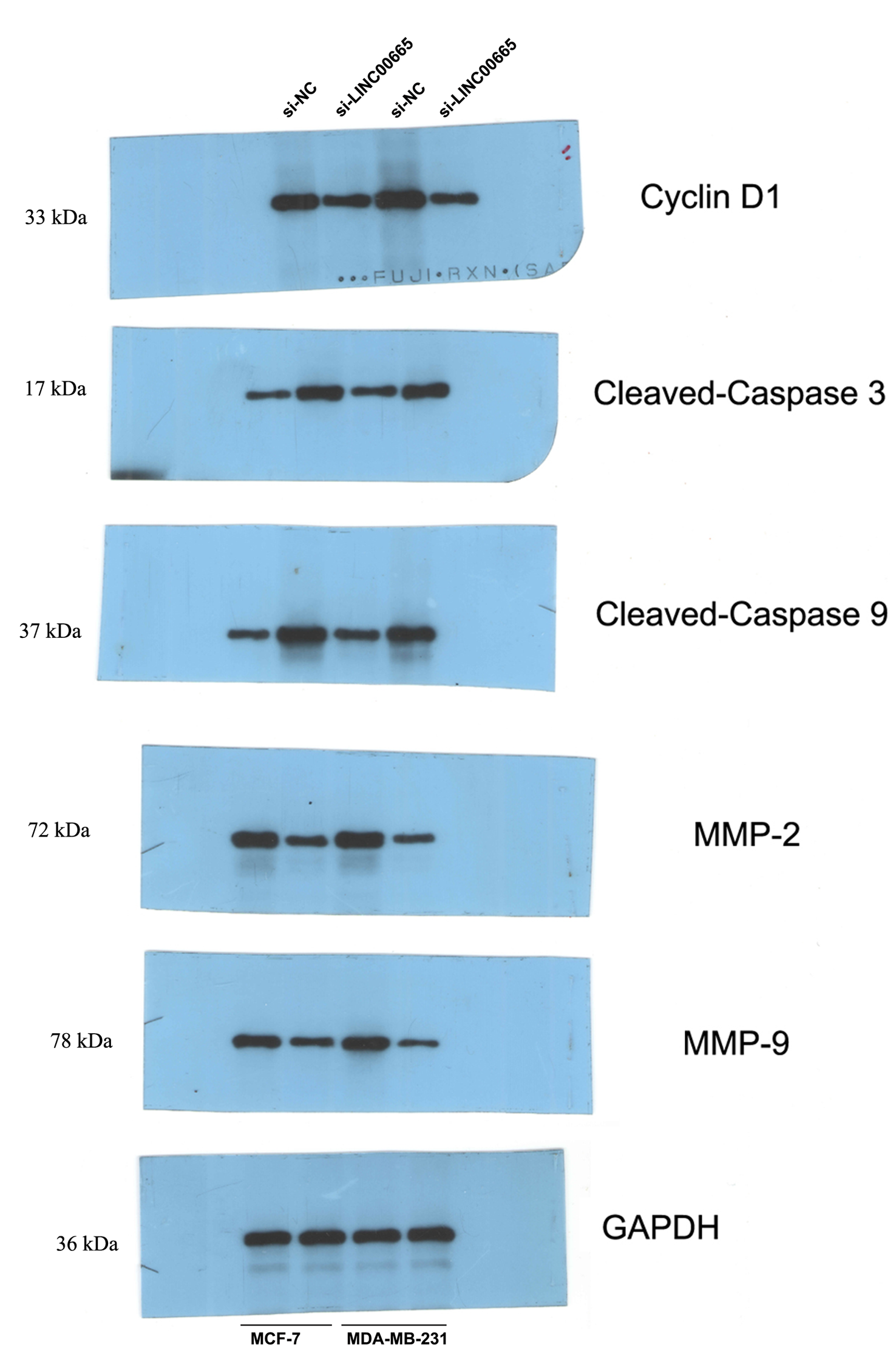

Supplement: Supplementary file 2 — Additional file 2: Figure S2. LINC00665 silencing reduced the protein expression levels of cyclin D1, matrix metalloproteinase (MMP)-2, and MMP-9 expression, while enhancing cleaved caspase-3 and cleaved caspase-9 expression, as measured by western blot analysis. [file 11658_2020_235_MOESM2_ESM.zip › WB-Figure S2.tif]

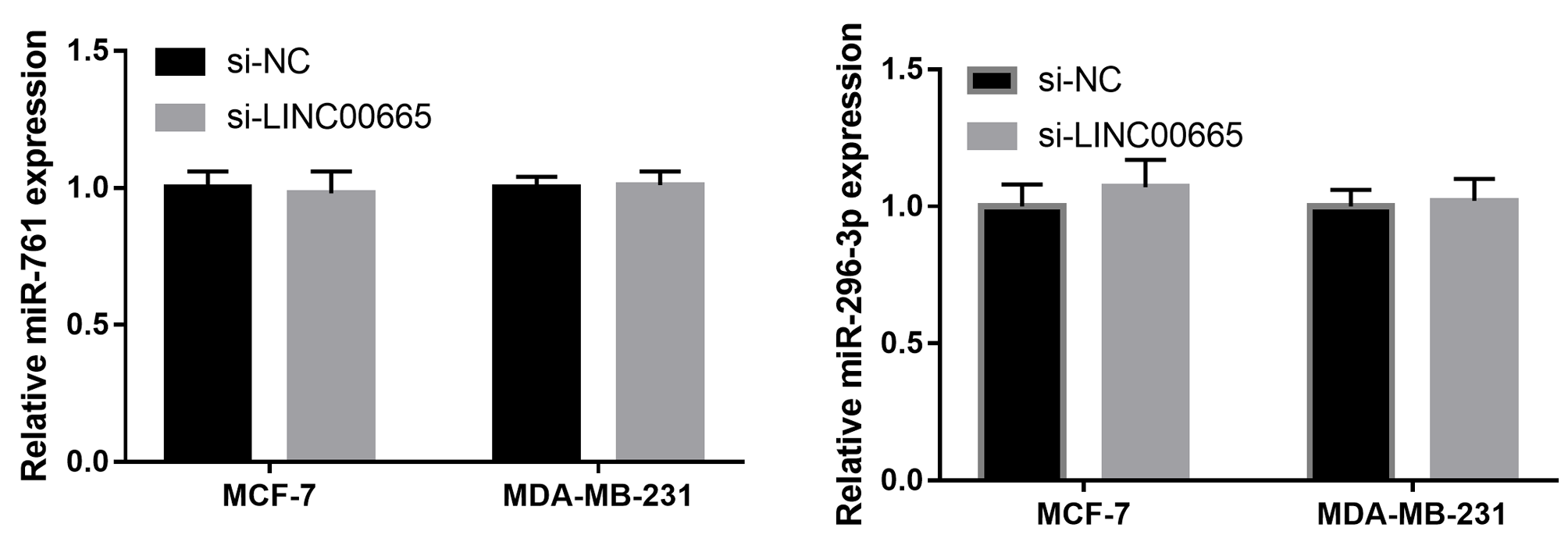

Supplement: Supplementary file 3 — Additional file 3: Figure S3. LINC00665 silencing did not significantly affect miR-761 and miR-296-3p expression. miR-761 and miR-296-3p expression, as measured by quantitative reverse transcription-polymerase chain reaction (qRT-PCR) analysis at 48 h after transfection with the si-LINC00665 plasmid. [file 11658_2020_235_MOESM3_ESM.tif]

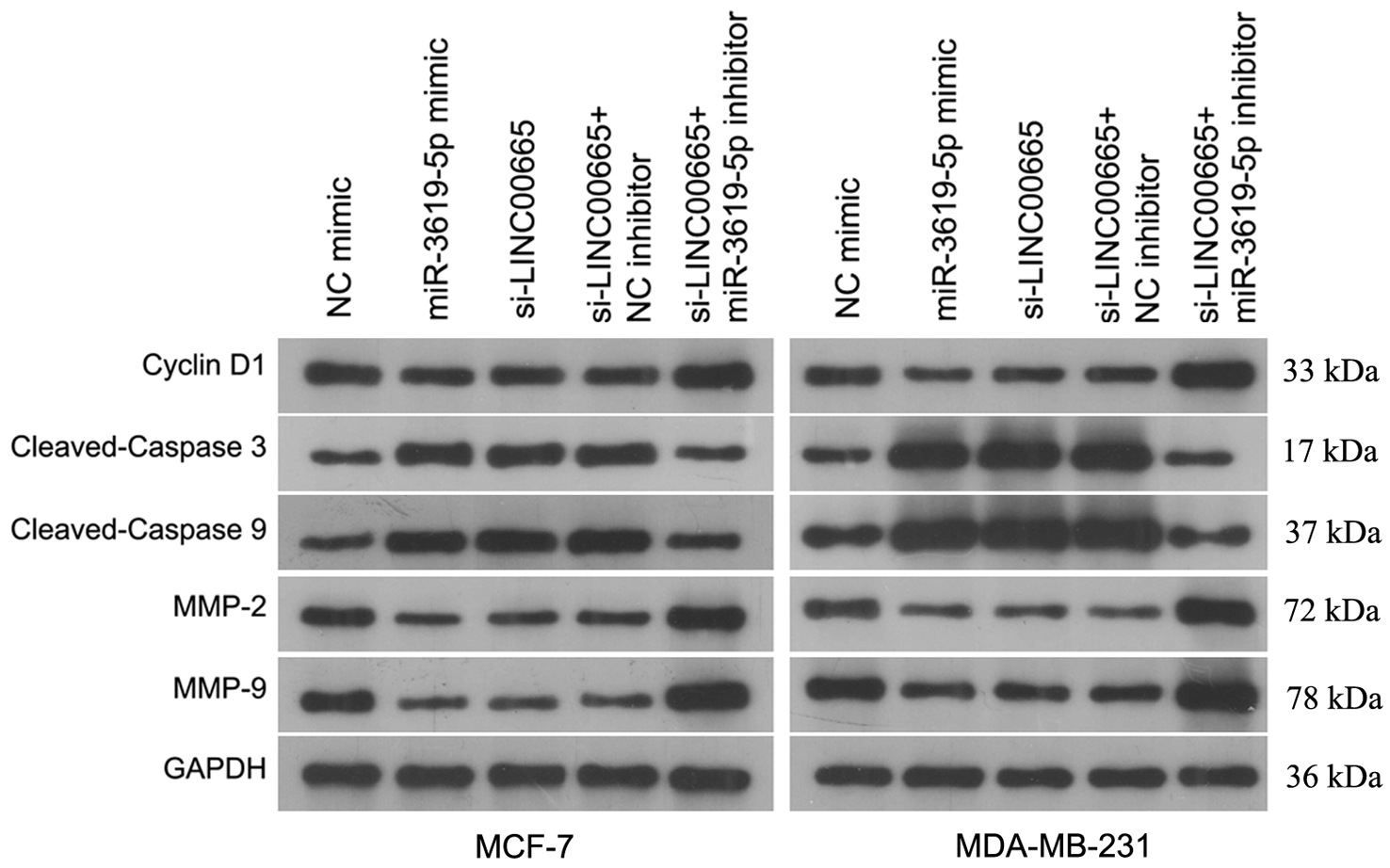

Supplement: Supplementary file 4 — Additional file 4: Figure S4. Effects of miR-3619-5p and LINC00665 on expression levels of cyclin D1, matrix metalloproteinase (MMP)-2, MMP-9, cleaved caspase-3, and cleaved caspase-9. The protein expression levels were measured by performing western blot analysis. [file 11658_2020_235_MOESM4_ESM.zip › R2-Figure S4.tif]

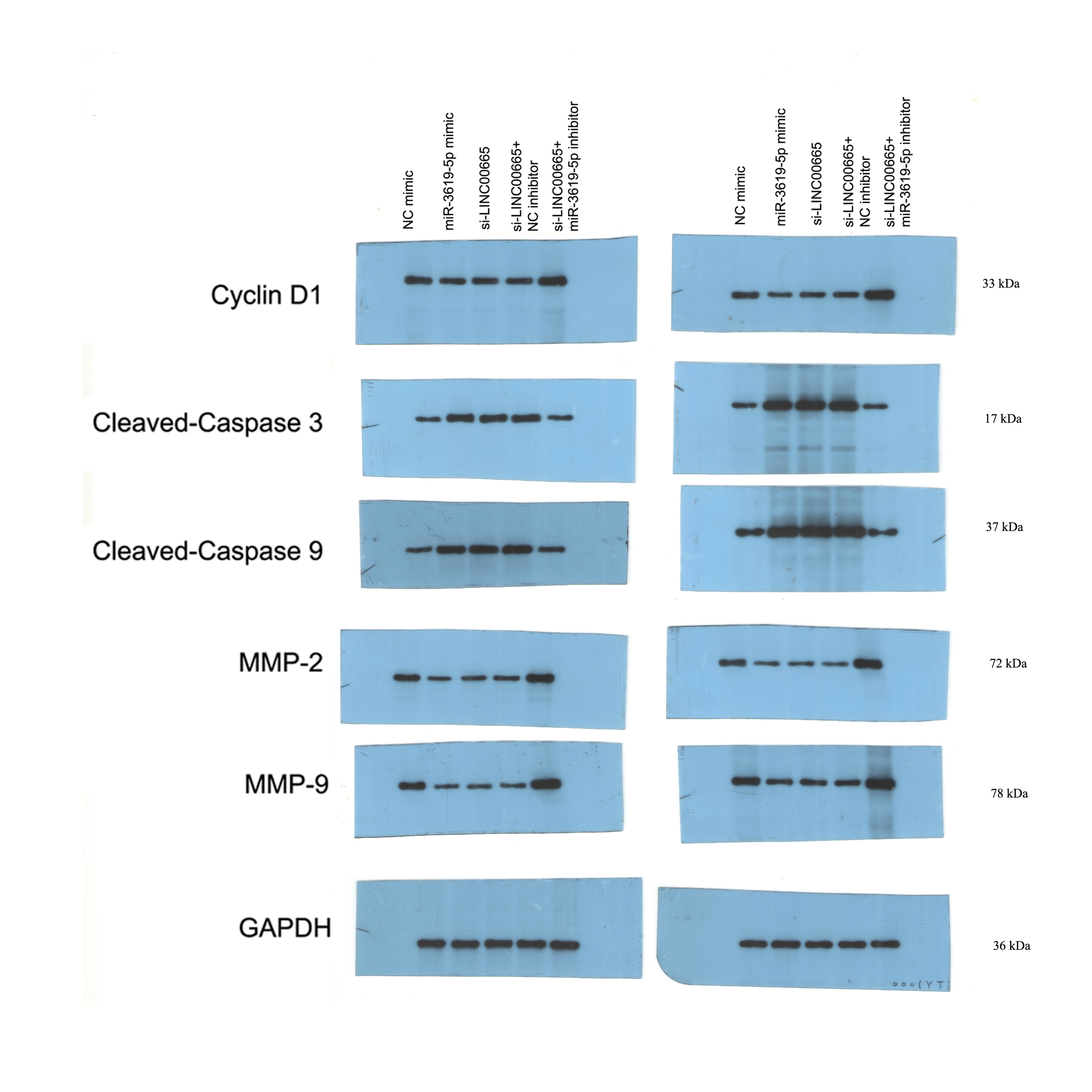

Supplement: Supplementary file 4 — Additional file 4: Figure S4. Effects of miR-3619-5p and LINC00665 on expression levels of cyclin D1, matrix metalloproteinase (MMP)-2, MMP-9, cleaved caspase-3, and cleaved caspase-9. The protein expression levels were measured by performing western blot analysis. [file 11658_2020_235_MOESM4_ESM.zip › WB-Figure S4.tif]
